# Epidemiological trends and incidence prediction of lung cancer in China based on the Global Burden of Disease study 2019

**DOI:** 10.3389/fmed.2022.969487

**Published:** 2022-09-20

**Authors:** Han Li, Meng Zhao, Gaoqiang Fei, Zemin Wang, Shuai Wang, Pingmin Wei, Wei Li

**Affiliations:** ^1^Department of Epidemiology and Health Statistics, School of Public Health, Southeast University, Nanjing, China; ^2^Department of Quality Management, Children's Hospital of Nanjing Medical University, Nanjing, China

**Keywords:** lung cancer, China, epidemiological trends, prediction, Global Burden of Disease study

## Abstract

Lung cancer remains the most common malignancy in China. This study aims to provide scientific support for the prevention and treatment of lung cancer by analyzing the epidemiological trends of lung cancer in China from 1990 to 2019. Based on the global health exchange database (GHDx), joinpoint and age-period-cohort analyses were performed to explore the trend of lung cancer incidence and mortality rates from 1990 to 2019. According to incidence rates from 1990 to 2019, a model was constructed to predict the incidence rates in the next 5 years. In addition, changes in risk factors associated with lung cancer deaths were compared between 1990 and 2019 and between males and females in 2019. The results are as follows. The age-standardized incidence rates (ASIRs), and age-standardized death rates (ASDRs) of lung cancer among Chinese had overall upward trends from 1990 to 2019. The ASDRs of females and males in China decreased since 2010. Interestingly, from 2016 to 2019, the ASIRs and ASDRs of females rose significantly. The age-period-cohort model showed that the incidence and mortality rates of lung cancer in China increased with age, and the growth rate accelerated after 45 years old. After 2004, the relative risks of lung cancer incidence increased with the passage of the period. Also, after the 1950–1954 birth cohort, the risks of lung cancer incidence and death began to decrease. The autoregressive integrated moving average (ARIMA) model predicted that the incidence rates of lung cancer in China would continue to rise in the next 5 years. The top five risk factors for lung cancer deaths of both genders in 2019 were smoking, ambient particulate matter pollution, secondhand smoke, high fasting plasma glucose, and household air pollution from solid fuels. The above results provided precise clues for the prevention and treatment of lung cancer in China.

## Introduction

According to the World Cancer Report 2020, there have been 2.2 million new cases of lung cancer worldwide, and deaths due to lung cancer will account for 18% of all deaths from cancer worldwide (1). Based on cancer statistics in China in 2016, lung cancer ranked first in terms of new cases (828,000) and deaths (657,000). Among males, lung cancer ranked first in the incidence and mortality rates of malignant tumors, while lung cancer ranked second in the incidence rate and first in the mortality rate among females from 2000 to 2016 (2). Previous study indicated that China serves one of the countries with the highest tracheal, bronchus, and lung cancer disease burden (3). There is no doubt that lung cancer has become a major public health problem and a huge social burden in China. It is of great significance to continue to carry out epidemiological research on lung cancer and strengthen the prevention and treatment of lung cancer to reduce the incidence and mortality of lung cancer in China. Lifestyle, environmental and occupational exposures are common risk factors for lung cancer (4), and understanding the deaths of lung cancer due to these risk factors is essential for disease management.

The Global Burden of Disease (GBD) project aggregated 354 diseases and injuries from 195 countries and territories worldwide, making it possible to analyze epidemiological trends of lung cancer in China (5). Therefore, based on the latest data published by the GBD project, the epidemiological trends from 1990 to 2019 in lung cancer in China were analyzed. Joinpoint and age-period-cohort models were developed based on incidence and death data to comprehensively analyze the epidemiological characteristics. Based on the incidence rates data, autoregressive integrated moving average (ARIMA) and support vector machine (SVM) models were used to construct models and finally the optimal model was selected to predict the incidence rates of lung cancer in the next 5 years. Additionally, the attributable risk factors for lung cancer deaths were also explored. The above analysis results might provide clues to the precise prevention and control of lung cancer.

## Materials and methods

### Data source

Incidence, deaths, and their age-standardized rates of lung cancer were downloaded from the GBD project (http://ghdx.healthdata.org/gbd-results-tool). In addition, 87 types of detailed risks were selected for identifying attributable risk factors for lung cancer deaths. By applying the comparative risk assessment (CRA) theory and counterfactual analysis, the proportions of lung cancer deaths caused by the above factors in the target population were calculated, that is, the population attributable score (PAF) of the risk factors (6).

### Joinpoint regression analysis

Joinpoint regression is a widely used statistical method to analyze the long-term trends of incidence or mortality rates of tumors and chronic diseases. The core idea of the model is to build segmental regressions based on the temporal characteristics of the disease distribution, and then to evaluate the characteristics of disease variability specific to different intervals in more detail over the global time horizon (7). In this study, based on age-standardized incidence rates (ASIRs) and age-standardized death rates (ASDRs), joinpoint regression models were constructed by Joinpoint Regression Program (4.9.0.0) to analyze global and local change trends in incidence and mortality rates of lung cancer. The annual percentage change (APC) [(*e*^β^−1) × 100%] and average annual percentage change (AAPC) [(*e*^∑ω*iβi*/∑ω*i*^−1) × 100%] are used to describe the direction and magnitude of the changing trend, where β is the regression coefficient, and ωi is the number of years included in each segment. When APC or AAPC > 0, it means that the incidence or mortality rates of lung cancer show a rising trend. When APC or AAPC < 0, it means the incidence or mortality rates show a decreasing trend. *P* < 0.05 represents a statistically significant change.

### Age-period-cohort analysis

The age-period-cohort model is based on Poisson distribution and uses three parameters: age, period, and birth cohort to reflect changes in the risks of disease incidence and death in the target population. And to quantify the effects of age, period, and cohort on incidence and death data based on controlling for interaction effects (8). In this study, an online tool provided by the National Cancer Institute (NCI) (http://analysistools.nci.nih.gov/apc/) was used to execute age-period-cohort analysis for the incidence and death of lung cancer (9). The tool performs the analysis using a built-in algorithm and the corresponding Wald test, with main parameters: net drift, local drifts, longitudinal age curve, period rate ratio, and cohort rate ratio. 15 age groups (20–24, 25–29, ……, 85–89, 90–94), 6-period groups (1990–1994, 1995–1999, ……, 2015–2019), and 20 cohorts (1900–1904, 1905–1909, ……, 1995–1999) were included in age-period-cohort models.

### Incidence prediction models

ARIMA time series model (p, d, q) which consists of an autoregressive model (AR) and a moving average model (MA) model have good accuracy and effectiveness in predicting the trend of future development of events, with p and q being the orders of AR and the order of MA, and d is the numbers of difference in stabilizing the term trend process (10). SPSS 25 was applied for ARIMA modeling in the study. SVM serves as a machine learning method that can be used for classification and regression prediction. A linear function was employed to predict the regression in a large space (11). In this study, the SVM model was developed through Python. ARIMA and SVM models were developed using the ASIRs of lung cancer in China from 1990 to 2014. Subsequently, the ASIRs of 2015–2019 were used to evaluate the predictive performance of the models. The evaluation indicators include mean square error (MSE) ($\frac{1}{n}\overset{n}{\underset{i=1}{\sum }}{\left(yt-ŷt\right)}^{2}$), mean absolute error (MAE) ($\frac{1}{n}\sum |yt-ŷt|$), and mean absolute percentage error (MAPE) ($\frac{1}{n}\sum \left(\frac{yt-ŷt}{yt}\right)\times 100\%$), where yt is the actual value, ŷt is the predicted value, and n is the number of predicted data.

## Results

### The general trends of incidence and mortality rates of lung cancer in China

**Figure 1:**
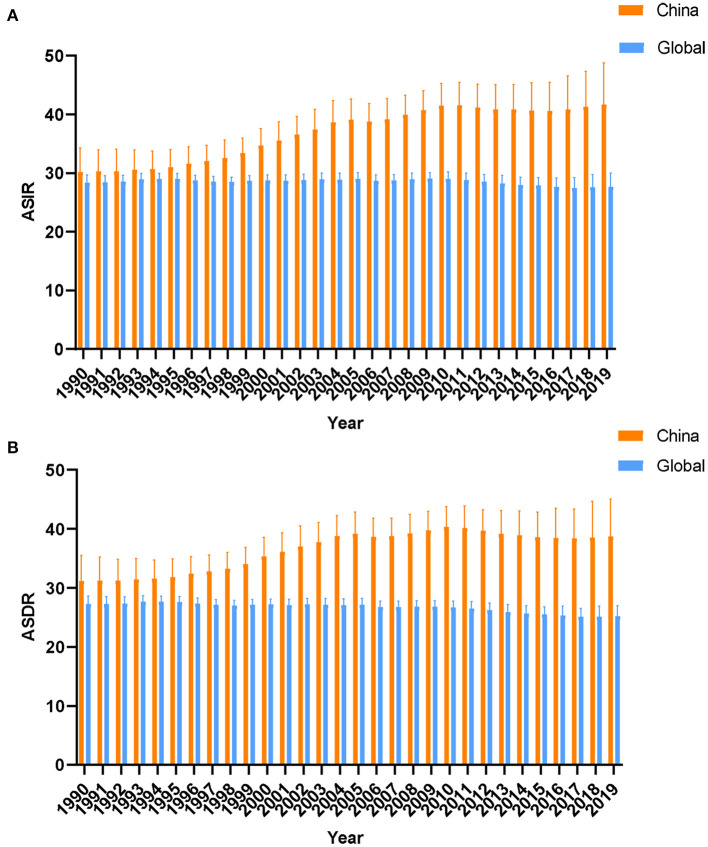
The trends of age-standard incidence rates **(A)** death rates **(B)** of lung cancer in global and China from 1990 to 2019.

From 1990 to 2019, the ASIRs of lung cancer in China showed an overall upward trend, with the highest in 2019 (Figure 1A). Figure 1B shows that the ASDRs of lung cancer in China increased overall from 1990 to 2010, with the highest in 2010. Since 2010, the ASDRs showed a slight downward trend. In general, the ASIRs and ASDRs of lung cancer in China were all higher than the global level.

### Description of segmental trends in lung cancer incidence and mortality rates from 1990 to 2019

**Figure 2:**
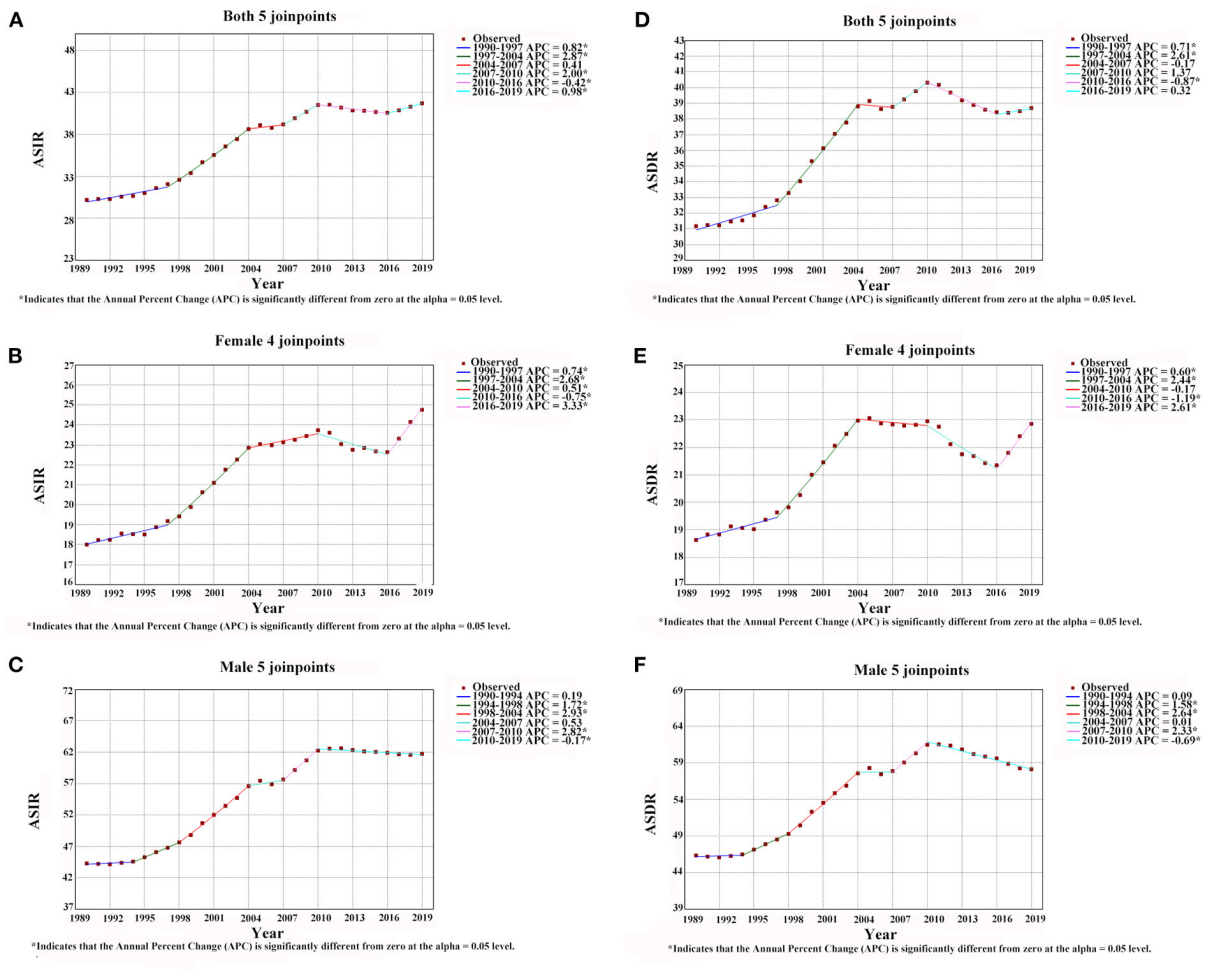
The results of joinpoint regression analysis for age-standard incidence rates and mortality rates of lung cancer in Chinese males [**(C)** incidence rate, **(F)** mortality rate], females [**(B)** incidence rate, **(E)** mortality rate] and both genders [**(A)** incidence rate, **(D)** mortality rate] from 1990 to 2019.

According to joinpoint analysis, ASIRs and ASDRs of lung cancer were on the rise for both males and females in China from 1990 to 2019 (All AAPC > 0, *P* < 0.001) (Table 1). ASIRs and ASDRs for both sexes increased by an average of 1.1 and 0.8% per year, respectively. In addition, Figure 2 shows that the ASIRs and ASDRs of both sexes and females of lung cancer had upward trends during 1990–1997, 1997–2004 (All APC > 0, *P* < 0.05), whereas it showed a downward trend during 2010–2016 (APC < 0, *P* < 0.05). For males, ASIRs and ASDRs of lung cancer increased from 1994 to 1998, 1998 to 2004, and 2007 to 2010 (All APC > 0, *P* < 0.05), while decreased from 2010 to 2019 (APC < 0, *P* < 0.05). Interestingly, it can be observed that ASIRs and ASDRs of females showed a clear upward trend in 2016–2019, with APCs of 3.33 and 2.61, respectively.

**Table 1:** Lung cancer incidence and mortality rates by sex in China, 1990–2019.

**Year**	**ASIR**			**ASDR**		
	**Female**	**Male**	**Both**	**Female**	**Male**	**Both**
1990	18.01	44.29	30.20	18.63	46.33	31.18
1991	18.23	44.21	30.30	18.83	46.18	31.24
1992	18.25	44.15	30.30	18.83	46.08	31.22
1993	18.56	44.38	30.58	19.12	46.26	31.47
1994	18.53	44.58	30.67	19.07	46.47	31.54
1995	18.51	45.28	31.01	19.02	47.14	31.85
1996	18.88	46.09	31.60	19.37	47.87	32.40
1997	19.18	46.78	32.08	19.63	48.51	32.83
1998	19.42	47.64	32.59	19.82	49.30	33.27
1999	19.89	48.81	33.38	20.26	50.44	34.03
2000	20.63	50.72	34.69	21.01	52.29	35.32
2001	21.10	52.02	35.55	21.46	53.54	36.13
2002	21.76	53.46	36.58	22.06	54.84	37.06
2003	22.27	54.71	37.44	22.49	55.87	37.78
2004	22.86	56.60	38.63	22.98	57.55	38.81
2005	23.04	57.47	39.10	23.06	58.26	39.15
2006	22.98	56.88	38.78	22.87	57.42	38.63
2007	23.13	57.65	39.19	22.84	57.84	38.78
2008	23.26	59.17	39.93	22.79	59.04	39.25
2009	23.45	60.73	40.70	22.82	60.27	39.77
2010	23.73	62.27	41.51	22.95	61.44	40.32
2011	23.61	62.57	41.53	22.75	61.51	40.18
2012	23.04	62.62	41.19	22.11	61.33	39.68
2013	22.76	62.36	40.86	21.75	60.82	39.19
2014	22.85	62.14	40.82	21.69	60.17	38.89
2015	22.67	62.06	40.68	21.43	59.84	38.60
2016	22.64	61.91	40.58	21.35	59.59	38.44
2017	23.31	61.69	40.88	21.80	58.84	38.40
2018	24.15	61.58	41.30	22.40	58.23	38.49
2019	24.76	61.74	41.71	22.86	58.10	38.70
AAPC(%)	1.1	1.2	1.1	0.7	0.8	0.8
95%CI	1	1	0.9	0.6	0.6	0.5
	1.3	1.3	1.4	0.9	1	1
*P* value	<0.001	<0.001	<0.001	<0.001	<0.001	<0.001

CI, confidence intervals.

### The age, period, and cohort effects on lung cancer incidence and mortality rates

**Figure 3:**
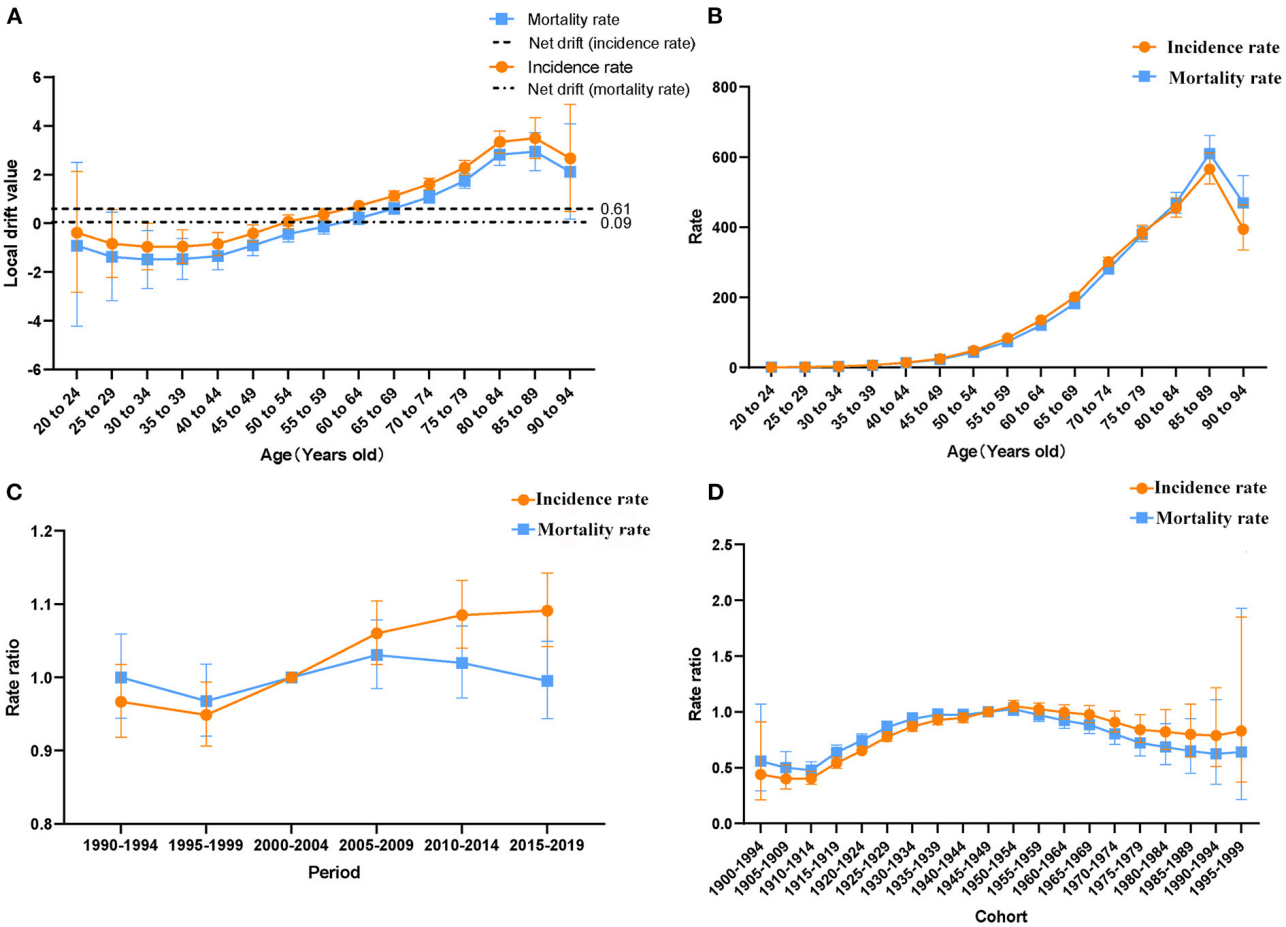
Age-period-cohort model. **(A)** Net drift and local drifts of incidence and mortality rates of lung cancer in China from 1990 to 2019. **(B)** Longitudinal age curve of lung cancer incidence and mortality rates in China. **(C)** Period relative risks of lung cancer incidence and mortality rates in China **(D)** Cohort relative risks of lung cancer incidence and mortality rates in China.

The wald test showed that the net drift, local drifts, period, and cohort effects of lung cancer incidence rates from 1990 to 2019 were all statistically significant (*P* < 0.01). However, for mortality rates of lung cancer, the local drifts and cohort effect were statistically significant (*P* < 0.01), while the net drift and period effect were not statistically significant (Table 2). For incidence rates, the net drift which represents the overall annual percentage change was 0.61% (95% confidence interval, CI: 0.38 to 0.84%), and the local drifts which represent additional age-specific variations were < 0 before the 50–54 age group and >0 after that. Additionally, after the 60–64 age group, the local drifts of mortality rates started to be >0 (Figure 3A). This showed that the incidence rates of each age group were increasing over time, with an average increase of 0.61% per year. And the incidence rates after the 50–54 age group and mortality rates after the 60–64 age group showed an increasing trend with time, with the rest showing a decreasing trend.

**Table 2:** Wald Chi Square tests for estimable functions in the age-period-cohort model.

**Null hypothesis**	**ASIR**		**ASDR**	
	**χ2**	***P***	**χ2**	***P***
Net Drift = 0	26.63	<0.01	0.44	0.51
All age deviations = 0	1177.89	<0.01	722.44	<0.01
All period deviations = 0	8.67	0.07	7.71	0.10
All cohort deviations = 0	230.93	<0.01	197.77	<0.01
All period RR = 1	34.99	<0.01	7.89	0.16
All cohort RR = 1	439.96	<0.01	265.16	<0.01
All local drifts = Net Drift	225.55	<0.01	193.50	<0.01

As shown in Figure 3B, after correcting for period and cohort effects, the longitudinal age curves of lung cancer incidence and mortality rates in China were upward trends before the age group of 85–89. The incidence and mortality rates were at a low level in the 20–44 age group, and they increased rapidly after the 45–49 age group, reaching the peak in the 85–89 age group. As for the period effect (Figure 3C), the period-related RRs of incidence rates were on the rise from the 2000–2004 period group (RR = 1) to the 2015–2019 period group. Concerning cohort effects (Figure 3D), after adjusting the effects of period and age, the cohort risks of incidence and mortality rates were a single-peaked distribution, with the cohort risks of incidence and mortality reaching their highest in the 1950–1954 cohort (RR = 1.05, 95% CI: 1.00 to 1.10, RR = 1.02, 95% CI: 0.97 to 1.08).

### Changes in attributed risk factors

**Figure 4:**
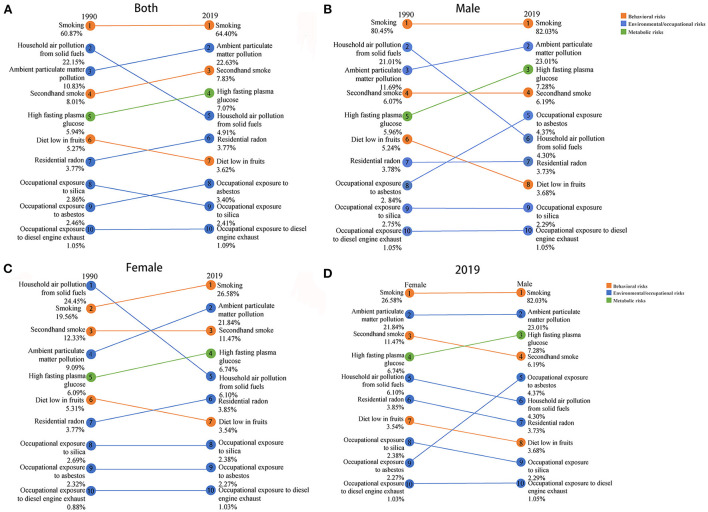
Comparisons of attributed risk factors of deaths between 1990 and 2019 in both genders **(A)** and males **(B)**, and females **(C)**, and between females and males in 2019 **(D)**.

As shown in Figure 4A, in 1990, smoking, household air pollution from solid fuels, and ambient particulate matter pollution ranked the top three contributors to the deaths of lung cancer in China, causing 60.87%, 22.15%, and 10.83% of deaths, respectively. In 2019, the top three contributors to lung cancer deaths were smoking, ambient particulate matter pollution, and secondhand smoke, which contributed to 64.40%, 22.63%, and 7.83% of deaths, respectively. Among them, household air pollution from solid fuels dropped from the 2nd (22.15%) in 1990 to the 5th (4.91%) in 2019. And diet low in fruits and occupational exposure to silica dropped one place. However, the attribution ranking of ambient particulate matter pollution, secondhand smoke, high fasting plasma glucose, residential radon, and occupational exposure to asbestos was slightly up. As for changing trends of the risk factors subdivided by gender (Figures 4B,C), the attribution ranking of ambient particulate matter pollution and high fasting plasma glucose rose in males and females from 1990 to 2019. And the attribution ranking of household air pollution from solid fuels dropped from 1990 to 2019. Additionally, deaths of lung cancer in females for smoking increased from 19.56% to 26.58%. Furthermore, as shown in Figure 4D, the top three risk factors for lung cancer deaths in Chinese males in 2019 were smoking (82.03%), ambient particulate matter pollution (23.01%), and high fasting plasma glucose (7.28%). Nevertheless, smoking (26.58%), ambient particulate matter pollution (21.84%), and secondhand smoke (11.47%) ranked as the top three risk factors for the death of lung cancer in Chinese females in 2019. High fasting plasma glucose and occupational exposure to asbestos had a greater impact on lung cancer deaths in males than in females. In addition, secondhand smoke, household air pollution from solid fuels, residential radon, a diet low in fruits, and occupational exposure to silica had a slightly greater impact on lung cancer deaths in females than in males.

### The prediction for lung cancer incidence in China in the future

Through the expert modeler of SPSS, the optimal model of ARIMA was determined to be (0, 2, 0). The Ljung-BOX test (*P* = 0.99 > 0.05) proved the constructed model was the white noise sequence. In addition, the SVM model was developed with kernel = “rbf”, degree = 3, gamma = “auto”, and C = 1. Table 3 shows the comparison of actual and predicted incidence rates in 2015–2019 of the two models. By comparing the goodness of fit of the two models (Table 4), the ARIMA (0, 2, 0) model with a better fitting effect was finally selected to predict the incidence rates of lung cancer in China in the next 5 years (2020–2024). The prediction results showed that the incidence rates of lung cancer in China will continue to rise in the next 5 years and might reach 44.10 /100,000 in 2024 (Table 5).

**Table 3:** Comparison of actual and predicted incidence rates between ARIMA and SVM models.

**Year**	**Actual value** ** (1/100,000)**	**Predicted value (1/100,000)**	
		**ARIMA**	**SVM**
2015	40.68	40.78	40.07
2016	40.58	40.73	39.60
2017	40.88	40.69	39.07
2018	41.30	40.66	38.52
2019	41.71	40.65	38.00

**Table 4:** Evaluation of the fitting effects of the two models.

**Evaluation indicators**	**ARIMA**	**SVM**
MAE	0.43	1.98
MSE	0.32	5.22
MAPE	0.0005	0.05

**Table 5:** The predicted values of ARIMA (0, 2, 0) model.

**Year**	**Predicted value (1/10,0000)**	**95%CI**
2020	42.14	41.29–43.00
2021	42.59	40.68–44.55
2022	43.06	39.88–46.43
2023	43.56	38.91–48.62
2024	44.10	37.83–51.14

## Discussion

With the rapid development of the economy, the lifestyle and disease spectrum of Chinese residents have changed dramatically. Among causes of death in China, lung cancer rose from the 8th in 1990 to the 4th in 2017. And as regards DALY (Disability-Adjusted Life Year) causes, lung cancer rose from 14th in 1990 to 4th in 2017 (12). Our study also indicated that lung cancer ASIRs and ASDRs increased overall in China from 1990 to 2019. Furthermore, joinpoint analysis indicated that ASDRs of females and males in China decreased since 2010. It is speculated that this might benefit from the initiation of low-dose computed tomography (LDCT) screening in 2010 in China (13). For the first time, the national lung screening trial research team demonstrated that LDCT screening reduced lung cancer mortality by 20% compared to chest X-ray (CXR) screening (14). The analysis results of this study also confirmed the necessity for early screening. Additionally, from 2016 to 2019, the ASIRs and ASDRs of females began to rise significantly again, with a rate of 3.33 and 2.61%, respectively. Cancer statistics in 2016 showed that for females, lung cancer was the second most common after breast cancer (2). Another epidemiological study in China showed that the average annual growth rate of lung cancer in women was greater than that in men from 2010 to 2015 (15, 16). In this study, it is found that the number of lung cancer deaths of females for smoking increased. This may be due to the rise in smoking rates in females in recent years. Moreover, lung cancer deaths from ambient particulate matter pollution increased as females work outside the home in recent years. Therefore, it is particularly significant to control these risk factors of lung cancer in females.

During the occurrence and development of diseases, individual characteristics such as age, birth cohort, and objective factors such as the social development period are all possible influencing factors. The age-period-cohort model has been widely used in lung cancer, colorectal cancer, cervical cancer, and other cancers, which is beneficial to reflect their changing trends more comprehensively, to seek the initiating factors and reasonable explanations for the occurrence and development of these cancers (17–19). In this study, the results of age effect analysis showed that the incidence and mortality rates of lung cancer in China increased with age, and the growth speed of the incidence and mortality rates accelerated after 45 years old. According to an analysis of China's 2016 cancer statistics, lung cancer is the most common cancer in males over 45 years old and females over 60 years old (2). On the one hand, the reason might be that lung cancer is usually diagnosed at an advanced stage (20), resulting in a later age of diagnosis. On the other hand, the risk of incidence and death from lung cancer will increase due to the increase in underlying diseases and decline in physical fitness in the elderly (21), which is consistent with our findings. At present, China's aging situation is severe. In 2019, China's population over 65 years old accounted for about 11.5% of the total population (22). Thus, the incidence and death of lung cancer in the middle-aged and elderly population should be paid attention to. The period effect can be understood as the risks of incidence and mortality of diseases due to changes in natural conditions or social environment in a specific period. The period effect analysis in this paper pointed out that after 2004, the period effects of incidence rates increased over time. First of all, this might be related to the increased exposure of Chinese residents to risk factors caused by the rapid development of society and economy and the accelerated process of industrialization (23, 24). Then, with the living standards improvement of Chinese residents, the risk of lung cancer increased due to poor eating habits such as excessive intake of red meat (25), and low levels of vitamin D (26). What's more, it is speculated that this might be related to the increase in the number of patients diagnosed with lung cancer caused by the screening program for high-risk groups of lung cancer launched in 2010 (13). Furthermore, residents of different birth cohorts will experience different disease exposure risks due to the different social, economic, natural environments and other factors (27). In the present study, the cohort effects of incidence and mortality rates showed a unimodal distribution and peaked in the 1950–1954 birth cohort. From the birth cohort of 1914–1919 to the birth cohort of 1950–1954, the risks of incidence and mortality increased. The reason might be that the earlier generation is less educated and has poorer health literacy resulting in less awareness of risk factors and therefore increases risks of morbidity and mortality of lung cancer (28). However, after the 1950–1954 birth cohort, the risks of lung cancer morbidity and mortality began to decrease. For one thing, after the founding of new China, the health system has gradually improved, and more emphasis has been placed on the prevention and control of chronic diseases. For example, the gradual improvement of China's medical insurance system reduces the medical burden on residents to a certain extent and enables timely treatment of lung cancer (29). For another thing, the younger generation is highly educated and has a strong sense of self-protection and high health literacy (30). Taken together, the younger generation has a lower risk of lung cancer morbidity and mortality.

The attributable risk factors for lung cancer deaths in China from 1990 to 2019 have changed. Lung cancer deaths due to household air pollution from solid fuels dropped from second to fifth. This is consistent with the results of another study in China (31). It could be seen that with the development of the times, household fuels in China were cleaner, and the indoor air has been purified. However, lung cancer deaths caused by ambient particulate matter pollution increased from the third to the second. It could be seen that with the process of industrialization, outdoor air pollution is still an important factor in the burden of lung cancer. And studies have shown that when smoking and air pollution affect men at the same time, the risk of developing lung cancer will be greatly increased (32). Our study found that deaths of lung cancer from smoking were the first in both 1900 and 2019. And the ranks of secondhand smoke rose from fourth to third. It means that the tobacco control policy in China has not worked well (14, 15). Also, deaths of lung cancer from high fasting plasma glucose increased. Hyperglycemia may accelerate cancer cell proliferation and is an independent predictor of survival in non-small cell lung cancer patients (33). Recently, eating habits have changed, which may explain this. In addition, the ranks of residential radon, and occupational exposure to asbestos all rose. Factors related to occupational exposure were also in the top ten. What's more, there were also differences in risk factors for lung cancer deaths in males and females in 2019. It's worth noting that occupational exposure to asbestos had a greater impact on lung cancer deaths in males than in females. The reason is that men have more exposure to asbestos in the workplace. Earlier research showed that men exposed to asbestos had a higher risk of developing lung cancer compared to men not exposed to asbestos (34). And females have longer exposure to household fuels and residential radon as they stay at home longer than males. Thus, household air pollution from solid fuels and residential radon had a greater impact on lung cancer deaths in females than in males. Based on the above understanding, the following suggestions are made: (1) Expanding the population of household clean fuel users, and encouraging the use of environmentally friendly decoration materials to purify indoor air. (2) Health education to improve public health literacy, combined with tobacco control policies, to reduce smoking rates. (3) Improving air quality while driving rapid economic growth. (4) Advancing diabetes screening and blood glucose monitoring. (5) Factors related to occupational exposure should also be paid attention to.

According to the forecast results of this study, the incidence rates of lung cancer in China might still increase in the next 5 years. Therefore, based on the analyses of epidemiological trends of lung cancer in China from 1990 to 2019 in the study, it can provide scientific reference for the prevention and control of lung cancer in the future. This study has the following limitations. Firstly, the incidence and death data from GBD are all estimates, and they are reconstructed from different sources. Secondly, this study only analyzes the epidemiological trends of lung cancer in the whole country, and cannot be specific to each province. Thirdly, in this study, the incidence rates of lung cancer were only predicted based on time series, but the occurrence of tumors is affected by many natural and social factors. In the future, more factors should be considered to optimize the model. Finally, the reported risk factors for GBD are limited, and more risk factors for lung cancer require further exploration.

## Conclusion

In summary, the incidence, and mortality rates of lung cancer among Chinese residents increased from 1990 to 2019. The results of the joinpoint analysis showed that from 2016 to 2019, the incidence and mortality rates of female lung cancer increased significantly, while the incidence and mortality rates of male lung cancer decreased significantly. The age-period-cohort model showed that the incidence and mortality rates of lung cancer in China increase with age, and the growth speed of incidence and mortality rates accelerated after 45 years old. After 2004, the period effect of incidence rate increased over time, and the period risk of mortality rate continued to decline after 2009. Also, after the 1950–1954 birth cohort, the risks of lung cancer morbidity and mortality began to decrease. Smoking, ambient particulate matter pollution, household air pollution from solid fuels, secondhand smoke, and high fasting plasma glucose remain major risk factors for lung cancer deaths. And based on the prediction in this study, the incidence rate of lung cancer will increase in the next 5 years. Therefore, based on the above results, in the prevention and control of lung cancer, it is necessary to pay more attention to women and middle-aged and elderly people, carry out early diagnosis and early treatment and combine risk factors for comprehensive prevention and control.

## Data availability statement

Publicly available datasets were analyzed in this study. This data can be found here: http://ghdx.healthdata.org/gbd-results-tool.

## Author contributions

HL was responsible for the study conception and design. SW was responsible for manuscript preparation. ZW contributed to data acquisition, analysis, and interpretation. MZ and WL contributed to the review of the data. PW and GF contributed to the critical revision of the manuscript, obtained funding, and supervised the research. All authors have read and approved the final manuscript.

## Funding

This work was supported by the Postgraduate Research & Practice Innovation Program of Jiangsu Province (KYCX22_0299).

## Conflict of interest

The authors declare that the research was conducted in the absence of any commercial or financial relationships that could be construed as a potential conflict of interest.

## Publisher's note

All claims expressed in this article are solely those of the authors and do not necessarily represent those of their affiliated organizations, or those of the publisher, the editors and the reviewers. Any product that may be evaluated in this article, or claim that may be made by its manufacturer, is not guaranteed or endorsed by the publisher.

## Abbreviations

GHDx: Global health exchange database
DALY: Disability-Adjusted Life Year
ASIR: Age-standardized incidence rate
ASDR: Age-standardized death rate
GBD: Global Burden of Disease
SVM: Support vector machine
APC: Annual percentage change
AAPC: Average annual percentage change
RR: Rate ratio
NCI: National Cancer Institute
AR: Autoregressive Model
MA: Moving Average model
MSE: Mean square error
MAE: Mean absolute error
MAPE: Mean absolute percentage error
CI: confidence intervals
ARIMA: Autoregressive Integrated Moving Average model
LDCT: Low-dose computed tomography
CXR: chest X-ray.
